# Serum levels of infliximab in Brazilian patients with Crohn's disease: what are the reasons for differences from previous studies?

**DOI:** 10.6061/clinics/2019/e1424

**Published:** 2019-09-19

**Authors:** Fabio Vieira Teixeira, Rogerio Serafim Parra, Omar Feres, Paulo Gustavo Kotze

**Affiliations:** I Clinica Gastrosaude, Marilia, SP, BR; II Faculdade de Medicina de Ribeirao Preto, Universidade de Sao Paulo, Ribeirão Preto, SP, BR; III Universidade Catolica do Parana (PUCPR), Curitiba, PR, BR

Dear Sir,

It was with significant interest that we read the manuscript, entitled“Serum Levels of Infliximab and Anti-Infliximab Antibodies in Brazilian Patients with Crohn's Disease”, recently published in this distinguished journal by Gomes et al. (1). In this study, the authors measured the serum infliximab (IFX) levels of 40 patients with Crohn's disease (CD) and correlated their findings with disease activity. There was no difference in the IFX level between patients with active disease and those with remission (*p*<0.05). Surprisingly, eighty percent of all patients had IFX levels above the therapeutic concentration (6-10 µg/mL).

Despite this cross-sectional study bringing interesting findings to the Brazilian literature, some important issues need to be explored. The therapeutic window considered by Gomes et al. was 6-10 µg/ml, which is different from what is considered globally, despite different assays being used worldwide. The TAXIT trial, one of the first prospective studies in the field, considered a therapeutic level of IFX for CD to be between 3 and 7 µg/mL (2). In an Australian consensus on therapeutic drug monitoring for CD, the therapeutic level for IFX was considered between 3 and 8 µg/mL (3). The definition considered by Gomes et al. might have mixed the results of the study, as several patients had levels from 3-6 µg/mL, which are considered adequate according to most studies but would be considered infra-therapeutic levels in the study from Campinas. If that remains an assay manufacturer's standard, this still needs clarification.

Even more surprising is the finding that the absolute majority of patients under IFX therapy in the study had supra-therapeutic levels (above 10 µg/mL). There have been 3 Brazilian manuscripts published to date regarding the serum infliximab levels in inflammatory bowel disease. The three studies considered a therapeutic IFX level of 3-7 µg/mL. The proportion of patients with supra-therapeutic levels was 17.46% according to Kampa et al. in a study using ELISA kits that were shipped and analyzed in Leuven, Belgium, which also included UC patients (4). Similar supra-therapeutic levels were found by Parra et al. (11.3%) in a study using the Quantum Blue rapid test (5). In another multicentric study, similar proportions were also found (8.16% with a different ELISA assay and 16.33% with the Quantum Blue rapid test) (6). The percentage of supra-therapeutic levels found by Gomes et al. (80% of the sample), even considering the upper limit of 10 µg/mL, is extremely high and not compatible with other studies from the same country. Most likely, different regimens of dose optimization or intrinsic differences among the different assays used could explain these findings, but that deserves clarification.

Another important point raised in the present study was that there was no difference in the serum level between patients with active disease and those in remission. This also goes in the opposite direction of the literature, which clearly demonstrates that higher levels of IFX are associated with higher rates of clinical, endoscopic and even histological remission (7). Possible reasons for the absence of a difference between the active and remission groups could be linked to the definition of disease activity, despite the authors using endoscopic and imaging tests to define remission.

The differences between the study by Gomes et al. and the national literature in this topic once more demonstrates the controversy that exists regarding therapeutic drug monitoring in the management of CD. Serum levels may vary according to assays, severity of the disease, albumin levels and other individual characteristics that are not yet known. Therefore, one should be careful in using serum levels as a single tool to optimize therapeutic management with IFX, by increasing doses, reducing intervals or even stopping therapy. A whole clinical picture of the patient needs to be considered before significant changes are made in practice.
